# Beyond *C9orf72*: repeat expansions and copy number variations as risk factors of amyotrophic lateral sclerosis across various populations

**DOI:** 10.1186/s12920-024-01807-9

**Published:** 2024-01-22

**Authors:** Zsófia Flóra Nagy, Margit Pál, József I. Engelhardt, Mária Judit Molnár, Péter Klivényi, Márta Széll

**Affiliations:** 1 Department of Medical Genetics, University of Szeged, Szeged, Hungary; 2 Department of Neurology, University of Szeged, Szeged, Hungary; 3 Institute of Genomic Medicine and Rare Disorders, Semmelweis University, Budapest, Hungary; 4 HUN-REN - SZTE Functional Clinical Genetics Research Group, Szeged, Hungary; 5 HUN-REN-SE Multiomics Neurodegeneration Research Group, Budapest, Hungary

**Keywords:** Amyotrophic lateral sclerosis, ALS, ALS genetics, Intermediate length repeat expansion, Copy number variation, *ATXN1*, *ATXN2*, *NIPA1*, *SMN1*, *SMN2*

## Abstract

Amyotrophic lateral sclerosis (ALS) is a neurodegenerative disorder which is characterized by the loss of both upper and lower motor neurons in the central nervous system. In a significant fraction of ALS cases - irrespective of family history- a genetic background may be identified. The genetic background of ALS shows a high variability from one ethnicity to another. The most frequent genetic cause of ALS is the repeat expansion of the *C9orf72* gene. With the emergence of next-generation sequencing techniques and copy number alteration calling tools the focus in ALS genetics has shifted from disease causing genes and mutations towards genetic susceptibility and risk factors.

In this review we aimed to summarize the most widely recognized and studied ALS linked repeat expansions and copy number variations other than the hexanucleotide repeat expansion in the *C9orf72* gene. We compare and contrast their involvement and phenotype modifying roles in ALS among different populations.

## Introduction

Amyotrophic lateral sclerosis (ALS) is a progressive, currently incurable neurodegenerative disease which leads to the degeneration of upper- and lower motor neurons [1]. The majority of the cases arise sporadically (sALS), while around 10–15% of the patients have a positive family history for the disease (fALS) [2].

The genetic background of ALS shows a great heterogeneity, around 130 genes with various inheritance patterns and penetrance have been associated with the development of the disease up to date [1]. The first gene to be linked to ALS was the superoxide dismutase 1 (*SOD1)* in 1993 [3]. The most important breakthrough in ALS genetics came in 2008 when two unrelated research groups identified an intronic hexanucleotide repeat element (GGGGCC) expansion of the Chromosome 9 open reading frame 72 gene (*C9orf72*) in ALS patients [4, 5].

In the last decade numerous studies aimed to uncover the role of repeat expansions other than *C9orf72* in ALS, such as *NIPA1*, *ATXN1* and *ATXN2*. Repeat expansions of these genes were primarily associated with other neurodegenerative conditions such as spinocerebellar ataxias and hereditary spastic paraplegia [6–8]. Furthermore, copy number variations (CNVs) in ALS have been in the focus of research in the recent years as well [9]. Excluding *C9orf72* repeat expansions, 17.6% of clinically diagnosed ALS and FTD cases had at least one expanded short tandem repeat (STR) allele reported to be pathogenic or intermediate for another neurodegenerative diseases such as *ATXN1* [spinal cerebellar ataxia type 1 (SCA1)], *ATXN2* (SCA2), *ATXN8* (SCA8), *TBP* (SCA17), *HTT* (Huntington’s disease), *DMPK* [myotonic dystrophy type 1 (DM1)], *CNBP* (DM2), and *FMR1* (fragile-X disorders) [10]. Our review focuses on the most extensively studied and most widely validated repeat expansions and copy number variations (Table 1) associated with ALS.

**Table 1:** Other repeat expansions associated with ALS up to date

Gene	Variant	Reference
*AR*	CAG repeat expansion	PMID: 2917278
*ATXN1*	intermediate length CAG expansion	PMID: 23197749
*ATXN2*	intermediate length CAG expansion	PMID: 20740007
*ATXN7*	CAG repeat expansion	PMID: 34870541
*ATXN8OS*	Noncoding repeat expansions	PMID: 30109267
*C9orf72*	GGGGCC	PMID: 21944778, PMID: 21944779
*CACNA1A*	CAG repeat expansion	PMID: 30342765
*CNBP*	CCTG repeat expansion	PMID: 37146135
*DMPK*	CTG repeat expansion in the 3′ UTR	PMID: 37146135
*FMR1*	CGG repeat expansion in the gene’s promoter region	PMID: 37146135
*FXN*	GAA repeat expansions	PMID: 24613765
*HTT*	CAG repeat expansion	PMID: 36285345
*LRP12*	CGG repeat expansion	PMID: 37339631
*NIPA1*	GCG repeat expansion	PMID: 22378146
*NOP56*	GGCCTG expansion	PMID: 35599735
*NOTCH2NLC*	GGC repeat expansion	PMID: 32989102
*STMN2*	CA repeat expansion	PMID: 33841129
*TBP*	CAG/CAA trinucleotide repeat expansions	PMID: 33377399

Within a single neuronal cell several pathogenic mechanisms may coexist, including RNA/protein toxic gain-of-function and/or protein loss-of-function. In case of *C9orf72* hexanucleotide repeat expansion the proposed pathogenic mechanisms can be both loss-of-function (LOF) and gain-of-function (GOF) mechanism. The formation of toxic dipeptide repeats and sequestration of RNA are characteristics of the GOF mechanism, while haploinsufficiency due to decreased expression from the altered *C9orf72* allele is a loss-of-function mechanism [11]. Out of the repeat expansions described in detail below *ATXN1* and *ATXN2* intermediate length trinucleotide repeat expansions are thought to be pathogenic by disrupting RNA processing mechanisms and by mislocalizing and sequestering important intracellular proteins [12, 13]. *NIPA1* polyalanine repeats are thought to change the secondary protein structure and increasing stability of interprotein bonds. Inclusion bodies similar to *ATXN1* and *ATXN2* intermediate length repeat expansion associated ALS cases have been observed in *NIPA1* repeat expansion carrier patients as well [14, 15]. The pathomechanism behind *SMN1* and *SMN2* copy number variations in connection to ALS remains elusive, several concepts were proposed. Overexpression of SMN proteins could be toxic to motor neurons, although there is no evidence that points this way. Another idea is that the copy number variation of the neighboring genomic region could be the key to understanding the role of *SMN* genes in ALS pathomechanism [16].

Genetics of ALS shows a great variability from one ethnicity to another which can only partly be explained by the different geographical locations of the populations [17]. Thus, it is of highest priority to genetically characterize ALS patients of different origins. In this review we aimed to summarize the current knowledge about the relationship of ALS and repeat expansions and CNVs in different genes from various ethnic groups.

### *C9orf72* repeat expansion

This is the most intensively studied repeat expansion in ALS. At the molecular level, the *C9orf72* GGGGCC repeat expansion has been linked to both frontotemporal dementia (FTD) and ALS [4, 5]. The expansion appears in approximately 25% of familial FTD patients and 20–67% of familial ALS patients depending on the population studied, making this the most prevalent genetic mutation in both diseases. The expansion also appears in approximately 6% of sporadic FTD patients and 7% of sporadic ALS patients [18].

Unaffected individuals have < 20 hexanucleotide repeats, while affected individuals have > 30 repeats and often even > 1000 repeats [4, 5]. The range of 20–30 repeats is considered to be an intermediate field with no clinical evidence of manifestation of either motor neuron disease or dementia. However, intermediate repeat lengths (20–30 repeats) have been reported to be a significant risk factor for developing Parkinson’s disease (PD) [19].

### *ATXN1* intermediate length repeat expansions

The presence of more than 44 CAG repeats in the *ATXN1* gene were identified as causative behind spinocerebellar ataxia 1 (SCA1) in 1993 [7]. *ATXN1* encodes ataxin-1, an RNA binding protein which has an important role in RNA metabolism [20]. According to experimental data the overexpression of ataxin-1 leads to the formation of both nuclear and cytoplasmic inclusion bodies. These inclusion bodies contribute to the mislocalization of TDP-43 (TAR DNA-binding protein 43) which is a core feature of ALS pathology [12].

The first paper exploring *ATXN1* repeat expansions in ALS was published in 2011, intermediate length alleles of the *ATXN2* gene were identified only a year after as a risk factor of ALS [13, 21]. However, in the 2011 study Lee et al., did not find a relationship between the two by testing more than 500 patients and the same number of controls [21].

The first report of *ATXN1* and ALS being connected came in 2012 when *Conforti* et al have found an association between ALS and *ATXN1* intermediate repeat lengths. The authors defined intermediate length alleles as more than 27 repeats but less than 44 CAG units. CAT triplet interruptions in the CAG repeat tract were also present in ALS patients just like in SCA1 patients. No clinical variables seemed to be linked to *ATXN1* intermediate repeat length alleles [22].

Subsequently *Lattante* et al., confirmed the link between ALS and intermediate length alleles of *ATXN1* (> 32). In their study, 9.16% of ALS patients and 5.48% of control individuals were positive for the intermediate repeat expansion. Interestingly, in more than one fifth of the *C9orf72* positive subgroup a concurrent *ATXN1* intermediate repeat expansion was detected as well. This finding highlights the oligogenic background of ALS and suggests a strong association of these two genes. A common pathology behind *C9orf72* and *ATXN1* genes cannot be excluded [23].

The link between *ATXN1* and the *C9orf72* repeat expansions was further explored in a paper published in 2020. 15.15% of sporadic ALS patients and almost twice this many familial ALS patients positive for the *C9orf72* expansion also carried *ATXN1* intermediate repeat expansions. The authors proposed that *ATXN1* is a disease modifier in *C9orf72* repeat expansion carriers and shifts the patients to develop ALS [24].

Among 182 investigated Hungarian ALS patients 8.79% carried the *ATXN1* intermediate allele compared to 1.12% of control individuals [25]. In the case of a female patient, who had a relatively late onset and fast progressing disease, co-harboring *ATXN1* and *C9orf72* repeat expansion was also described [25]. It could be proposed that the faster progression was due to the interplay between these two repeat expansions.

In a Maltese cohort describing 52 ALS patients, 17% of patients were identified to carry the intermediate repeat expansion (vs. 4.5% of healthy control individuals) [26]. This is the highest rate of *ATXN1* intermediate repeat expansion carriers reported, which may be due to the fact that the population of Malta is a relatively secluded island nation.

*ATXN1* intermediate alleles are also associated with ALS among Brazilian patients. 5.84% of ALS patients and 2.75% of control individuals carried an intermediate length allele longer than 33 CAG repeats. Frontotemporal dementia was also observed in 12.5% of *ATXN1* intermediate allele carrier ALS patients. However, the link between *C9orf72* and *ATXN1* could not be proved in this study [27].

Similarly to Brazil, the link between ALS and *ATXN1* intermediate repeat expansion could not be confirmed in African ALS patients and even the distribution of allele length significantly varied from European cohorts [28].

Reports on the co-occurrence of SCA1 and ALS have also been published. In a large SCA1 pedigree a male patient was described having a rapidly progressing motor neuron disease at the age of 47. *ATXN1* genotyping showed two intermediate length alleles without CAT triplet interruption. His brother, who developed ataxic symptoms at the age of 45, had a fully expanded *ATXN1* locus. At later stages of his diseases he showed ALS-like features as well (anarthria, dysphagia, fasciculations in the tongue) [29]. An another extended SCA1-ALS family was identified by targeted sequencing of ALS associated genes and CNV analysis. Furthermore, in this case a functional pathway analysis of the affected genes was also carried out. This revealed that dysregulation of synaptic transmission and lysosomal vesicular trafficking are important adversely altered processes in ALS patients and SCA1 patients with motor neuron signs [30].

The most comprehensive analysis on ALS and *ATXN1* up to date was published by *Tazelaar* et al [12]. More than 2600 ALS patients of different European countries were investigated. 12.2% (328/2672) of ALS patients and 10.1% (244/2416) of healthy individuals carried an intermediate allele in the *ATXN1* gene. The authors also performed a meta-analysis which concluded that ALS and *ATXN1* intermediate alleles are in fact linked to each other, however, *ATXN1* intermediate alleles do not influence either the survival or the age at onset [12]. Findings of a study on a model organism are also reported in that paper. Co-expression of *ATXN1* and *C9orf72* repeats resulted a more severe eye phenotype in *Drosophila melanogaster* compared to the expression of either of the *C9orf72* repeat expansion or the *ATXN1* repeat expansion [12]. This is a further evidence that the two repeat expansions aggravate the effect of each other in animal model, even though this phenotype modifying effect is yet to be confirmed in humans.

### *ATXN2* intermediate length repeat expansions

The *ATXN2* gene is responsible for coding the ataxin-2 protein which is involved in receptor trafficking and in modulation of endocytotic processes [31]. Upregulation of the fly-specific ataxin-2 protein in *Drosophila melanogaster* led to increased TDP-43 aggregation and toxicity which was seen in the eye of the fruit fly [13]. Data also suggests that the connection of ataxin-2 and TDP-43 is limited not only to yeast and *Drosophila*, but they also interact in human cells through the RNA recognition motif of the TAR DNA-binding protein 43 protein [13]. As the localization of the Ataxin-2, in healthy controls a dispersed pattern, also involving the nucleus, may be observed, while in ALS patients ataxin-2 is confined to intracytoplasmic accumulations in neurons [13]. Intermediate repeat expansion of the *ATXN2* gene increases the creation of reactive oxygen species by adversely influencing the function of NADPH oxidase [32]. A proposed pathomechanism behind *ATXN2* intermediate repeat expansion toxicity is the disruption of RNA metabolism and thus important RNA binding proteins may become sequestered [32]. Intermediate repeats are defined as more than 26 but less than 34 repeats. Alleles longer than 34 CAG triplets cause spinocerebellar ataxia 2 (SCA2) in an autosomal dominant manner [6].

The minimal cut-off number for intermediate length alleles was reviewed by multiple international research groups to increase the specificity and sensitivity. In 2010 Elden et al. first identified more than 26 CAG units in 4.7% of ALS patients and in 1.4% healthy individuals [13]. *Sproviero* et al.*,* and *Daoud* and colleagues independently defined intermediate repeats as more than 28 CAG units [33, 34]. An Italian study recommeneded > 30 trinucleotide units as a cut off value [35].

In the expanded *ATXN2* CAG tract CAA interruptions were identified in ALS patients [36, 37]. According to *Yu* et al., harboring at least 3 interruptions compared to less than three CAA triplets negatively influences the age at onset of the disease [37].

Families and patients co-exhibiting ALS and SCA2 symptoms have been abundantly reported [38–40]. A report from the UK described the case of a female patient who developed symptoms resembling SCA at the age of 67. Half a year later muscle wasting started which was accompanied by bulbar and upper motor neuron signs. Her *ATXN2* genotype was 33 repeats and a normal allele with 22 repeats and major ALS genes were screened without a relevant finding [41]. Two patients having the same *ATXN2* genotype (33/22 repeats) were described to initially present as either SCA2 or lower motor neuron sign dominant ALS. The patient presenting SCA2 later developed symptoms of ALS, his disease progressed rapidly and exited due to respiratory failure [38]. Heterozygous fully expanded alleles (39 repeats for the ALS patient and 40 repeats for the patient with SCA2) were also detected in SCA2 and ALS occurring in the same family in an uncle (ALS, 62 year old at onset, died 23 months after the onset of symptoms) and in his niece (SCA2, with 36 year old at onset) [40].

An Australian ALS patient was identified with the full length hexanucleotide expansion in the *C9orf72* gene, the intermediate repeat expansion in the *ATXN2* gene and the *NEK1* p.R261H variant [42]. His case also supports the polygenic nature of ALS. Two siblings of Guyanese ancestry who developed ataxia, parkinsonian symptoms and dementia were identified carrying both an expanded allele of *C9orf72* and *ATXN2* (37/22 repeats) [43]. Authors supposed that *ATXN2* intermediate expansions could contribute to both ataxic features and to the cognitive impairment and the interplay between these genes could lead to the emergence of the complex phenotype [43]. An animal study further supported this theory: Co-expressing *ATXN2* intermediate repeat expansion (30 repeats) and *C9orf72* hexanucleotide repeat expansion in zebra fish showed a synergic effect. Higher levels of *ATXN2* aggregation was observed and the fish exhibited aberrant swimming patterns and faulty axonal morphology [44].

Data of Russian ALS patients found a European-like percentage of *ATXN2* intermediate repeats (5% = 10/199 patients). However, these patients were exclusively recruited from the European region of Russia, so the results were up to the expectations [45]. In a study form Malta only one patient (4.17%) was found with a homozygous 28/28 intermediate length *ATXN2* allele, but this may be a consequence of the small sample size (1/24) [46]. Interestingly, patients from India also showed similar data to Europeans, as 4.6% (6/131 patients) of the investigated ALS patients carried an intermediate length allele (between 27 and 32 repeats) of the *ATXN2* gene [47]. The highest percentage of ALS patients carrying the intermediate repeat expansion has been reported from Brazil, where 6.3% (29/459 patients) of ALS patients were tested positive for *ATXN2* intermediate alleles [48]. Reports from China found a significantly lower rate of *ATXN2* intermediate alleles (1.6, 1.5 and 1.9%) among ALS patients as studies involving patients of European origin [49–51]. This comes as no surprise since the population of China vastly differs from European people as far as ancestry is concerned. 1.5% of ALS patients of Korean ancestry carried an intermediate allele [52]. Australian ALS patients also exhibit Asian-like numbers in terms of *ATXN2* intermediate alleles: Only 1.6% (10/616) of the patients harbored an intermediate length allele, which is unanticipated since Australia has seen vast number of European immigrants in the past, even though the investigated sample size is rather small [42]. *ATXN2* intermediate repeat expansions were seldom identified in African ALS patients and no link could be established between *ATXN2* and ALS [28].

Most studies agree that ALS patients with *ATXN2* intermediate repeat expansions do not differ in any demographical or clinical variable from patients without an *ATXN2* intermediate allele [13, 33, 47, 52]. However, a 2015 paper investigating 672 Italian ALS patients identified a significantly shorter survival in case of *ATXN2* positive patients compared to *ATXN2* negative patients (2.0 years vs. 3.2 years). Furthermore, a non-significant abundance of spinal onset ALS cases was observed among *ATXN2* intermediate allele carriers [35]. The same results with larger effect sizes were confirmed by a paper reporting on the genetic testing of 375 ALS patients from Sardinia [53].

It was recently reported by analyzing an ALS registry that *ATXN2* intermediate repeat expansions are not only associated with shorter survival and spinal onset but also with a faster time to diagnosis from onset of symptoms, faster decline of ALSFRS-R (Revised Amyotrophic Lateral Sclerosis Functional Rating Scale) score and with the presence of comorbid frontotemporal dementia [54]. It is noteworthy, that reports of *ATXN2* intermediate alleles being a phenotype modifier only stem from Italy. This could be explained by the fact that Italian researchers are very active in this field and perform detailed and comprehensive analyses.

### *NIPA1* GCG repeat expansion

The *NIPA1* gene codes the non-imprinted in Prader-Willi/Angelman syndrome region protein 1, a magnesium transporter that has an inhibitory role on the bone morphogenetic protein signaling pathway. This pathway is involved in the development of synapses and axons [55, 56] and *NIPA1* mutations have been identified in autosomal dominant spastic paraplegia [8].

In 2010 a CNV analysis study identified *NIPA1* rare deletion as a risk factor candidate for ALS [57]. Later the ALS associated role of *NIPA1* polymorphic GCG repeat expansions was confirmed in multiple European populations and longer alleles were associated with shorter survival of patients [58]: a large international cohort examining almost 4000 ALS patients found *NIPA1* alleles with more than 8 GCG units a risk factor of ALS with an odds ratio of 1.54 [59].

More rapid disease progression was also noted in a Maltese ALS patient with a heterozygous *NIPA1* expansion of more than 8 repeats [46]. A non-significant association between spinal onset, and earlier disease onset and *NIPA1* alleles of more than 8 repeats was also observed in two unrelated studies [60, 61].

The presence of *NIPA1* expansion was also investigated in a *C9orf72* positive subgroups of ALS patients [60, 61]. An Italian study did not find a significantly higher proportion of *NIPA1* expansion among *C9orf72* positive ALS patients (3.5% = 6 patients of *C9orf72* positive patients and 4.1% = 15 patients of *C9orf72* negative patients). Meanwhile 15.3% = 7 patients of *C9orf72* positive patients (46/755 patients, 6.1%) also carried a *NIPA1* expansion according to a paper from the Netherlands [60, 61]. As the results of the two studies are significantly diverse, further studies are needed to investigate the role of the joint expansion of *NIPA1* and *C9orf72* in ALS.

*NIPA1* repeats were shorter in populations with African origin compared to Europeans and did not show enrichment among African ALS patients [28].

### SMN1 and SMN2 genes

Homozygous deletions and compound heterozygous variants of the survival of motor neuron 1 (*SMN1)* gene cause spinal muscular atrophy (SMA) [62]. The severity of the SMA symptoms is mainly determined by the copy number of the highly homologous survival of motor neuron 2 (*SMN2)* gene [63].

The function of *SMN1* and *SMN2* genes in ALS is quite controversial. The first study linking ALS to *SMN1* and *SMN2* genes stems from 2001 [64]. In this report the authors found that homozygous deletions of the *SMN2* gene were 4 times more frequent in ALS patients than in controls (16% = 18/110 patients vs. 4% = 4/100 patients). In terms of clinical parameters, only the median time of survival seemed to differ, which was 1.9 year shorter in patients with *SMN2* homozygous deletions than in patients with 2 copies of *SMN2* [64]. A complete opposite finding was published by *Corcia* et al. In Swedish ALS patients with homozygous *SMN2* deletion the duration of the disease was more than 3 months longer compared to a formerly investigated unrelated cohort of French patients. Thereby, *SMN2* homozygous deletions were described to be protective in ALS [65].

A paper from 2002 described that both heterozygous deletions and heterozygous *SMN1* duplications are more common among ALS patients than in control subjects. Interestingly, spinal onset of ALS symptoms was quite common (71%) among ALS patients with one copy of *SMN1*. The authors proposed that the abnormal alleles could point to a linkage disequilibrium with the neuronal apoptosis inhibitor protein (*NAIP*) gene, which is located in close proximity to the *SMN1–2* locus [66].

In the subsequent years many replica studies were conducted. In 2005, a study from the Netherlands found that heterozygous *SMN1* deletion carriership was 3.8 times more frequent among ALS patients than in controls. Less copies of *SMN2* were also enriched in ALS patients. These data suggest that the total level of full-length wild type SMN protein level could be the key regarding ALS pathogenesis. Fewer *SMN2* copies and mortality rate showed a directly proportional relationship, while in case of fewer *SMN1* copies the correlation did not reach the level of significance [67].

In a large study in 2012, using firm methodology, three copies of the *SMN1* gene was found to increase susceptibility to ALS with an odds ratio of 2.07. The authors proposed that the heterozygous duplications of *SMN1* might play a more remarkable role than it was formerly indicated [16].

Although a meta-analysis from 2014 [68] confirmed the role of *SMN1* duplications as a risk factor of ALS, a recent bioinformatics study, using the DNA sequences of Project MinE (https://www.projectmine.com/) found no association between ALS and the copy number variations of either the *SMN1* or the *SMN2* gene [69].

In 25 Korean patients with motor neuron disease it was confirmed that *SMN2* homozygous deletions are a susceptibility factors for lower motor neuron disease. Later the association was also confirmed in sporadic ALS patients of Korean descent. Furthermore, ALS patients with homozygous *SMN2* deletions were found to have an earlier age at onset and a lower initial score on the Medical Research Council scale [70].

Since only European and Korean populations have been studied so far, there is a lack of population specific data regarding the connection of *SMN* genes and ALS. To settle the debate further studies, involving not yet studied populations, are needed. Moreover, the pathological processes in which copy number variations of *SMN1* and *SMN2* may be involved remain elusive. Designing functional studies and thus understanding the pathophysiology could bring solution to this two-decade-long debate.

## Conclusions

Amyotrophic lateral sclerosis is a diverse disease with defects of many genes implicated in diverse pathways contributing to faulty functioning of multiple mutually non-exclusive pathological processes. With the advent of high-throughput sequencing methods the focus of investigations has shifted from causal genes towards genetic risk factors in ALS genetics research. Uncovering the genetic background of ALS from disease causing genes to risk factors holds the key to understanding the pathomechanism of the disease in detail.

Although, based on a large genome scan study, rare CNVs with larger effect sizes do not play an essential role in ALS [57], investigating copy number variants in ALS might be prosperous. In other neurodegenerative diseases such as Alzheimer’s dementia and Parkinson’s disease the role of copy number variants has been extensively studied with convincing positive results [71, 72].

As we have summarized these genetic risk factors show variability from one population to another, thus characterization of different populations represents huge value. Even though there is not a huge phenotype modifying effect of any of the described ALS risk factors, according on the oligogenic disease theory of ALS, these small-effect repeat expansions and CNVs might be the final straw that broke the camel’s back and led to the emergence of the disease.

## Data Availability

Not applicable.
